# An Ovarian Reserve Assessment Model Based on Anti-Müllerian Hormone Levels, Follicle-Stimulating Hormone Levels, and Age: Retrospective Cohort Study

**DOI:** 10.2196/19096

**Published:** 2020-09-21

**Authors:** Huiyu Xu, Li Shi, Guoshuang Feng, Zhen Xiao, Lixue Chen, Rong Li, Jie Qiao

**Affiliations:** 1 Center for Reproductive Medicine Department of Obstetrics and Gynecology Peking University Third Hospital Beijing China; 2 Key Laboratory of Assisted Reproduction Ministry of Education Beijing China; 3 National Clinical Research Center for Obstetrics and Gynecology Beijing China; 4 Beijing Key Laboratory of Reproductive Endocrinology and Assisted Reproductive Technology Beijing China; 5 Big Data and Engineering Research Center Beijing Children’s Hospital Capital Medical University Beijing China; 6 Zhejiang Provincial People's Hospital Hangzhou China

**Keywords:** ovarian reserve, poor ovarian response, AMH, AFC, FSH, logistic regression

## Abstract

**Background:**

Previously, we reported a model for assessing ovarian reserves using 4 predictors: anti-Müllerian hormone (AMH) level, antral follicle count (AFC), follicle-stimulating hormone (FSH) level, and female age. This model is referred as the AAFA (anti-Müllerian hormone level–antral follicle count–follicle-stimulating hormone level–age) model.

**Objective:**

This study aims to explore the possibility of establishing a model for predicting ovarian reserves using only 3 factors: AMH level, FSH level, and age. The proposed model is referred to as the AFA (anti-Müllerian hormone level–follicle-stimulating hormone level–age) model.

**Methods:**

Oocytes from ovarian cycles stimulated by gonadotropin-releasing hormone antagonist were collected retrospectively at our reproductive center. Poor ovarian response (<5 oocytes retrieved) was defined as an outcome variable. The AFA model was built using a multivariable logistic regression analysis on data from 2017; data from 2018 were used to validate the performance of AFA model. Measurements of the area under the curve (AUC), sensitivity, specificity, positive predictive value, and negative predicative value were used to evaluate the performance of the model. To rank the ovarian reserves of the whole population, we ranked the subgroups according to the predicted probability of poor ovarian response and further divided the 60 subgroups into 4 clusters, A-D, according to cut-off values consistent with the AAFA model.

**Results:**

The AUCs of the AFA and AAFA models were similar for the same validation set, with values of 0.853 (95% CI 0.841-0.865) and 0.850 (95% CI 0.838-0.862), respectively. We further ranked the ovarian reserves according to their predicted probability of poor ovarian response, which was calculated using our AFA model. The actual incidences of poor ovarian response in groups from A-D in the AFA model were 0.037 (95% CI 0.029-0.046), 0.128 (95% CI 0.099-0.165), 0.294 (95% CI 0.250-0.341), and 0.624 (95% CI 0.577-0.669), respectively. The order of ovarian reserve from adequate to poor followed the order from A to D. The clinical pregnancy rate, live-birth rate, and specific differences in groups A-D were similar when predicted using the AFA and AAFA models.

**Conclusions:**

This AFA model for assessing the true ovarian reserve was more convenient, cost-effective, and objective than our original AAFA model.

## Introduction

The antral follicle count (AFC) is the number of follicles <8 mm in diameter in early gonadotropin-dependent follicular growth. It has been widely accepted that the pool of primordial follicles in the ovary—the ovarian reserve—is related to the number of growing antral follicles. Thus, in theory, the AFC reflects the remaining ovarian follicle pool [1-3]. However, obtaining an accurate AFC demands a time- and resource-consuming ultrasound examination by a skilled transvaginal sonography specialist. The lack of standardization in AFC measurements [4], AFC changes through the menstrual cycle, contraceptive use [5], and the sensitivity and resolution of transvaginal sonography equipment are all confounding factors making the reliable assessment of AFC difficult.

We have previously published a model for estimating ovarian reserves, using 4 predictors: anti-Müllerian hormone (AMH) level, the AFC, follicle-stimulating hormone (FSH) level, and age. This model was named as the AAFA (anti-Müllerian hormone level–antral follicle count–follicle-stimulating hormone level–age) model [6]. With the development of accurate AMH assays [7,8], the level of this hormone might replace the use of AFC in the measurement of ovarian reserve, avoiding the complexity, cost, and interobserver variation in the AFC [9,10]. Here, we aimed to explore the possibility of establishing a model for assessing a true ovarian reserve using the 3 predictors: AMH levels, FSH levels, and age. This model is referred to as the AFA (anti-Müllerian hormone level–follicle-stimulating hormone level–age) model. If the performance of the AFA model without using the AFC is only slightly worse or even similar to the 4-predictor AAFA model, it might be of better clinical significance, especially in physical examination centers or third-party clinical laboratories, which cannot perform AFC measurements by transvaginal sonography.

## Methods

### Subjects

This was a retrospective observational cohort study using the same dataset as in our previous study [6]. Briefly, data from 2017 to 2018 were selected according to the inclusion and exclusion criteria. In total, we selected 1523 oocytes from ovarian cycles stimulated by a gonadotropin-releasing hormone (GnRH) antagonist 2017 and 3273 oocytes, from 2018. The first and second stimulation cycles were included as described by Xu et al [6], and there were no strict restrictions on the women’s age or body mass index. Diseases potentially related to defects in follicular development were excluded, including ovarian cysts, previous ovarian surgery, polycystic ovarian syndrome, previous metabolic or endocrinological diseases, previous tuberculosis, chromosomal abnormalities, and women with pregnancies within the previous 3 months. The need for informed consent by the patients was waived, and institutional review board approval was not needed for the de-identified data in this retrospective analysis, as per the Declaration of Helsinki [11].

### Sampling and Endocrine Assays

Venous blood samples were drawn, and the sample tubes were immediately inverted 5 times to facilitate thorough blood clotting. Serum was collected by centrifugation and used for endocrine assessment. The circulating FSH level was measured on menstrual cycle day 2, and the circulating AMH level was measured on any day of the menstrual cycle. Serum FSH measurements were performed using a Siemens Immulite 2000 immunoassay system (Siemens Healthcare Diagnostics). The quality controls used for the FSH assay were Lypocheck Immunoassay Plus Control, Trilevel, catalog number 370, lot number 40340 (Bio-Rad Laboratories). Serum AMH concentrations were measured by an ultrasensitive 2-site enzyme-linked immunosorbent assay (Ansh Laboratories), using quality controls supplied within the kits. The coefficients of variation for each assay were indicated previously [6].

### Statistical Analysis

In this study, poor ovarian response with <5 oocytes retrieved was defined as an outcome variable. The predictor variables were age and basal serum FSH and AMH concentrations. A multivariable logistic regression analysis was performed to construct a predictive model for poor ovarian response to stimulation using 2017 data; the data from 2018 were used to validate the performance of that model. Measurements of the area under the curve (AUC), sensitivity, specificity, positive predictive value (PPV), and negative predicative value (NPV) were used to evaluate the predictive models. The main effect of each predicting variable measures the variation over the distribution of *x*_j_ in the mean poor ovarian response. Venn diagrams were used to compare the differences between the AAFA and AFA models.

To rank the ovarian reserve of the whole population, we ranked subgroups according to the predicted probability of poor ovarian response and further divided the 60 subgroups into 4 groups A-D, according to cut-off values consistent with our established AAFA model [6]. Analyses were conducted using SAS JMP Pro (version 14.2; SAS Institute), and *P*<.05 was considered statistically significant.

## Results

We previously established an AAFA model, using the 4 predictors of AMH, AFC, FSH, and age [6]. We used this to classify the study population into 4 subgroups: A, B, C and D.

However, the lack of standardization in AFC measurements [4] makes the reliable assessment of AFC very difficult. Moreover, the AFCs were well correlated with AMH, FSH, or age [12,13], implying collinearity. Therefore, we sought to explore the possibility of establishing a model for assessment of the true ovarian reserve using only 3 predictors—AMH, FSH, and age (ie, the AFA model)—instead of the previous 4-predictor AAFA model [6].

### Multivariable Logistic Regression to Build a Predictive Model for Poor Ovarian Response Using the 2017 Data

Basic characteristics of the treatment cycles are shown in Table 1.

As in our previous study, we transformed the 3 continuous variables of age, AMH, and FSH into categorical variables. The data used here were exactly the same as those from 2017, when we built our AAFA model [6]. The cut-off values of each predictor in both AFA and AAFA models are listed in Table 2.

**Table 1:** Basic characteristics of treatment cycles.

Characteristics	2017 (n=1523)	2018 (n=3273)
Age (years), Mean (SD)	33.4 (5.3)	32.7 (4.8)
Basal FSH^a^ (IU/L), Mean (SD)	7.5 (3.3)	7.2 (3.1)
AMH^b^ (ng/mL), Median (IQR)	2.2 (1.1-4.0)	2.7 (1.2-4.8)

^a^FSH: follicle-stimulating hormone.

^b^AMH: anti-Müllerian hormone.

**Table 2:** Comparison of grouping criteria of the AFA and AAFA models.

Grouping criteria	AFA^a^ model groups						AAFA^b^ model groups	
	0	1	2	3	4	0		1
AMH^c^ (ng/mL)	<0.5	0.5 to <1	1 to <1.5	1.5 to <2	≥2	≥1.2		<1.2
Basal FSH^d^ (IU/L)	<6.5	6.5 to <8.5	8.5 to <10.5	≥10.5	N/A	≤8		>8
Age (years)	≤30	>30 to 40	>40	N/A	N/A	≤35		>35
AFC^e^	N/A	N/A	N/A	N/A	N/A	≥8		<8

^a^AFA: Anti-Müllerian hormone level–Follicle-stimulating hormone level–Age.

^b^AAFA: Anti-Müllerian hormone level–Antral follicle count–Follicle-stimulating hormone level–Age.

^c^AMH: anti-Müllerian hormone.

^d^FSH: follicle-stimulating hormone.

^e^AFC: antral follicle count.

The transformed categorical variables were then analyzed using multivariable logistic regression. The main effects that each independent variable exerted in this model were AMH (85.2%), followed by FSH (6.8%), and age (2.8%). Thus, we have named this model as AFA based on the order of the main effects of each predictor. The odds ratios of each predictor are indicated in Table 3.

**Table 3:** The odd ratios of each predictor in the AFA model.

Predictors	OR (95% CI)	*P* value
Intercept	1.753 (1.177-2.611)	<.001
Categorical age (1 vs 0)	2.239 (1.344-3.731)	.006
Categorical age (2 vs 0)	1.863 (1.270-2.734)	.002
Categorical FSH^a^ (1 vs 0)	2.300 (1.438-3.681)	.002
Categorical FSH (2 vs 0)	3.594 (2.333-5.538)	.001
Categorical FSH (3 vs 0)	24.641 (14.997-40.488)	<.001
Categorical AMH^b^ (0 vs 4)	11.431 (7.281-17.945)	<.001
Categorical AMH (1 vs 4)	5.010 (3.167-7.928)	<.001
Categorical AMH (2 vs 4)	2.211 (1.259-3.882)	<.001
Categorical AMH (3 vs 4)	1.753 (1.177-2.611)	.006

^a^FSH: follicle-stimulating hormone.

^b^AMH: anti-Müllerian hormone.

### Comparing the Performances of the AFA and AAFA Models

To further evaluate the performance of this AFA model, we calculated the AUC, sensitivity, specificity, PPV, and NPV in the training set (2017 data) and the validation set (2018 data) as indicated in Table 4.

A calibration plot was drawn to evaluate the calibration performance of the AFA model in the training set and validation set (Multimedia Appendix 1). The performance of the AAFA model in the validation set (2018 data) is indicated in Table 4. A comparison shows that the AUCs of the AFA and AAFA models for the same validation set are similar at 0.853 (95% CI 0.841-0.865) and 0.850 (95% CI 0.838-0.862), respectively. The specificity, sensitivity, PPV, and NPV are also indicated in Table 4. The AUC between AFA model and AAFA model was tested with DeLong test. The difference of the 2 models in AUC level is 0.009 (95% CI –0.004 to 0.022), indicating no significant difference.

**Table 4:** The performance of AFA model.

Performance indicators	AFA^a^ model		AAFA^b^ model
	Training set, OR (95% CI)	Validation set, OR (95% CI)	Validation set, OR (95% CI)
AUC^c^	0.860 (0.843-0.877)	0.853 (0.841-0.865)	0.850 (0.838-0.862)
Sensitivity	0.511 (0.456-0.566)	0.489 (0.445-0.533)	0.519 (0.475-0.563)
Specificity	0.940 (0.925-0.952)	0.949 (0.940-0.957)	0.930 (0.920-0.939)
PPV^d^	0.688 (0.626-0.744)	0.633 (0.585-0.680)	0.570 (0.525-0.615)
NPV^e^	0.881 (0.862-0.897)	0.911 (0.901-0.922)	0.915 (0.904-0.925)

^a^AFA: Anti-Müllerian hormone level–Follicle-stimulating hormone level–Age.

^b^AAFA: Anti-Müllerian hormone level–Antral follicle count–Follicle-stimulating hormone level–Age.

^c^AUC: area under the curve.

^d^PPV: positive predictive value.

^e^NPV: negative predictive value.

**Figure 1:**
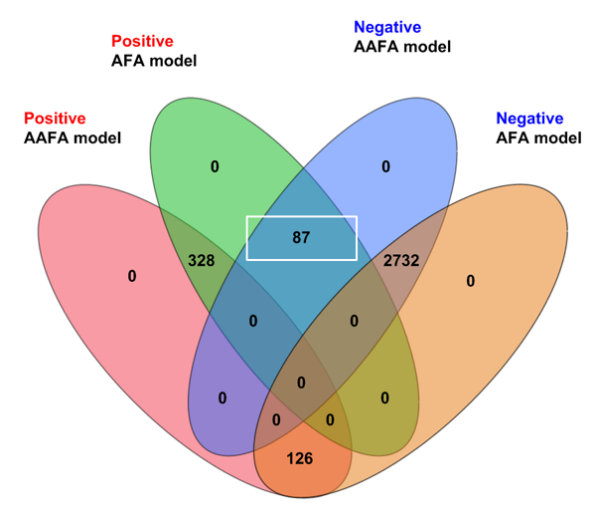
Comparison of the performances of the AFA and AAFA models in the 2018 validation data.

The numbers of overlapping and nonoverlapping cases in the predicted positive (poor ovarian response) and negative estimated by the 2 models are shown in Figure 1.

There were 328 positive (poor responders) and 2732 negative (nonpoor responders) cases overlapping in the 2 models. The 2 models had 93.5% (3060/3273) overlapping positive and negative cases in the 2018 validation set.

### Ranking the Ovarian Reserve Based on the Predicted Probability of a Poor Ovarian Response

We previously ranked the ovarian reserve of the whole population according to the predicted probability of a poor ovarian response [6], given that the number of oocytes retrieved is closely related to the number of primordial follicles in the ovarian cortex [14-16]. In this study, we used the same method to rank the ovarian reserve according to the predicted probability of a poor ovarian response calculated using the AFA model. The 60 groups were further divided into 4 subgroups: A, B, C, and D (Multimedia Appendix 2).

The order of ovarian reserve from adequate to poor followed the order of predicted probability of a poor ovarian response from low to high. Women with a predicted probability of more than 50% were classified into the population with diminished ovarian reserve (namely, group D that includes subgroups 43-60), as shown in Multimedia Appendix 2. The actual incidences of poor ovarian response, clinical pregnancy rate per starting cycle, clinical pregnancy rate per embryo transfer cycle, live-birth rate per starting cycle, and live-birth rate per embryo transfer cycle (with 95% CIs) are also indicated in Table 5.

**Table 5:** The clinical pregnancy rate and live-birth rate in the 4 ovarian reserve groups.

Ovarian reserve group and model		Actual incidence of poor ovarian response (95% CI)		CP^a^ per starting Cycles (95% CI)		CP per ET^b^ cycles (95% CI)		LB^c^ per starting Cycles (95% CI)		LB per ET cycles (95% CI)	
**A**											
	AFA^d^		0.037 (0.029-0.046)		0.217 (0.199-0.235)		0.454 (0.424-0.486)		0.176 (0.160-0.193)		0.368 (0.339-0.399)
	AAFA^e^		0.038 (0.030-0.046)		0.212 (0.195-0.229)		0.439 (0.409-0.469)		0.174 (0.158-0.190)		0.360 (0.331-0.388)
**B**											
	AFA		0.128 (0.099-0.165)		0.165 (0.123-0.205)		0.298 (0.242-0.361)		0.138 (0.108-0.175)		0.249 (0.197-0.309)
	AAFA		0.139 (0.101-0.177)		0.210 (0.166-0.254)		0.370 (0.300-0.439)		0.167 (0.126-0.207)		0.294 (0.228-0.359)
**C**											
	AFA		0.294 (0.250-0.341)		0.157 (0.124-0.197)		0.277 (0.221-0.340)		0.142 (0.110-0.180)		0.249 (0.196-0.310)
	AAFA		0.362 (0.308-0.415)		0.140 (0.101-0.179)		0.402 (0.309-0.495)		0.124 (0.087-0.161)		0.355 (0.265-0.446)
**D**											
	AFA		0.624 (0.577-0.669)		0.135 (0.105-0.171)		0.289 (0.229-0.356)		0.080 (0.057-0.110)		0.170 (0.124-0.229)
	AAFA		0.571 (0.525-0.616)		0.126 (0.095-0.156)		0.268 (0.208-0.327)		0.077 (0.053-0.102)		0.164 (0.114-0.213)

^a^CP: clinical pregnancy.

^b^ET: embryo transfer.

^c^LB: live birth.

^d^AFA: Anti-Müllerian hormone level–Follicle-stimulating hormone level–Age.

^e^AAFA: Anti-Müllerian hormone level–Antral follicle count–Follicle-stimulating hormone level–Age.

### Comparing Specific Differences Between the AFA and AAFA Models in Groups A-D

**Figure 2:**
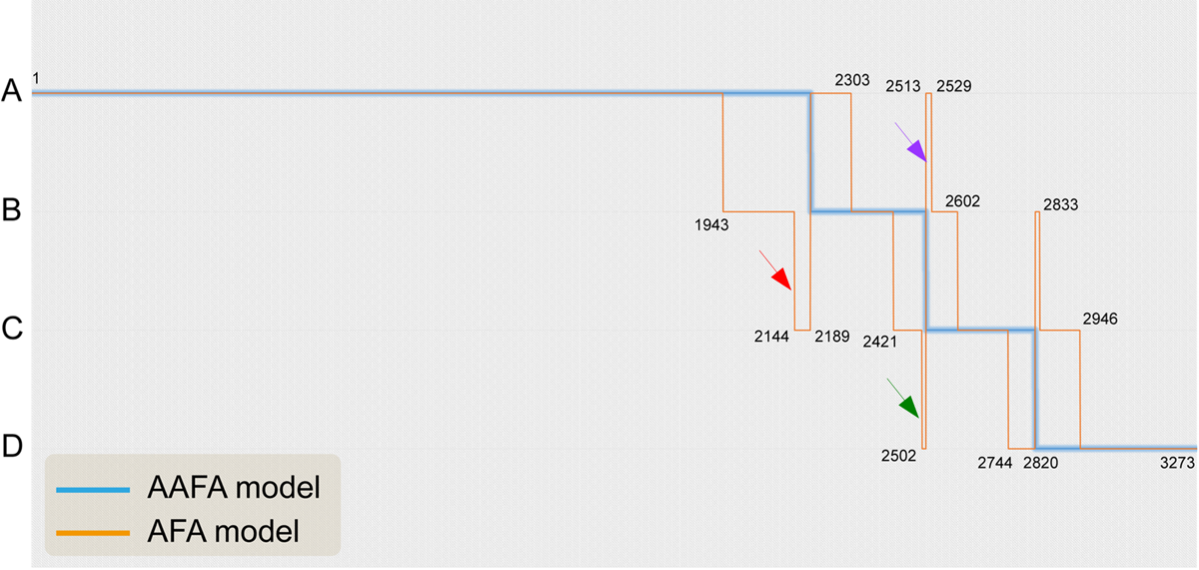
Specific differences between the AAFA and AFA models in A-D groups.

Figure 2 displays the specific differences between the AFA and AAFA models in classifying the whole population into groups A-D.

**Figure 3:**
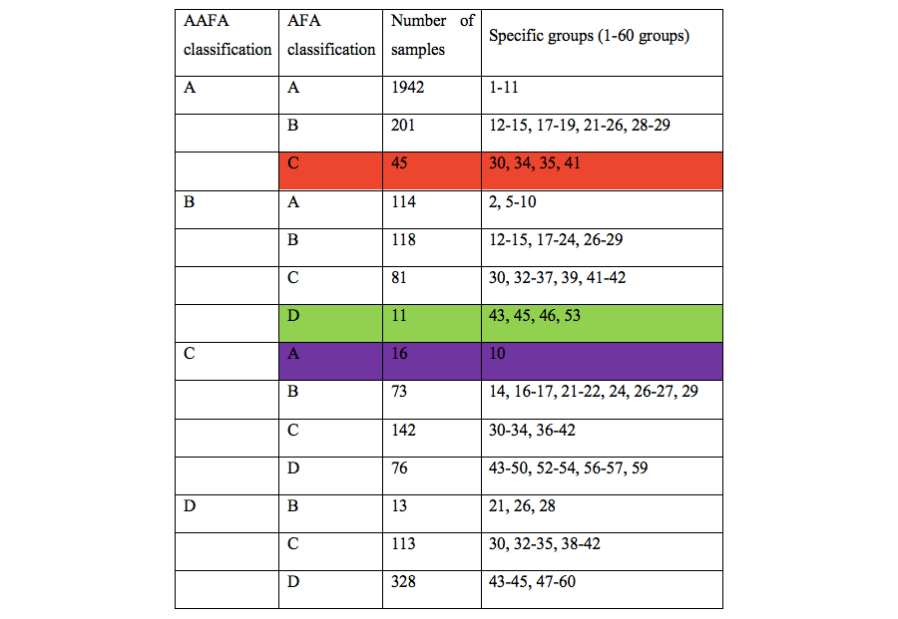
Specific differences between the AAFA and AFA models in A-D groups (tabular form).

The horizontal axis includes the 3273 cases in the 2018 validation data. The 2 models did not show a 3-level difference; that is, there was no case classified as A (good ovarian reserve) by the AAFA model but as D (diminished ovarian reserve) by the AFA model. In addition, most cases were classified into the same groups by both models. However, there were differences for some cases. We focus on 3 groups having 2-level differences defined by the AFA or AAFA models, as shown by the red, green, and purple arrows in Figure 2. The same colors are used to indicate those 3 groups in Figure 3.

The raw data and the corresponding predicted probability of a poor ovarian response in the 2 models are listed in Multimedia Appendix 3. The actual incidences of poor ovarian response in the 3 subgroups were 4/45 (red), 5/11 (green), and 1/16 (brown). These results suggest that for the red subgroup, the AAFA classification might be closer to the actual incidence of poor ovarian response (4/45). Thus, these cases should have been placed in group A, rather than in the group C. However, for the purple subgroup with a poor ovarian response incidence of 1/16, the group A classified by AFA model might be more suitable. For the green subgroup with a poor ovarian response incidence of 5/11, not group B by the AAFA model or group D by the AFA model, but group C is more appropriate with its predicted probability of 30% to 50%. For groups having 1-level differences, specific cohorts are shown in Figure 2, Figure 3, and Multimedia Appendix 3.

## Discussion

We previously established our AAFA model to assess ovarian reserve based on AMH, AFC, FSH, and age [6]. However, standardization of the AFC has long been difficult for fertility clinics worldwide. In this study, using the same 2018 validation data without the AFC predictor, the AFA model showed similar performance as that of the AAFA model, with an AUC of 0.853 (95% CI 0.841-0.865) vs 0.850 (95% CI 0.838-0.862) for the AAFA model. Since it does not require the AFC, the applicability and cost-effectiveness of the AFA model is better than the AAFA model. Thus, a large number of first- and second-tier hospitals, physical examination centers, or third-party clinical laboratories, which cannot conduct AFC tests, can now assess ovarian reserve using our AFA model.

There were no large (3-level) differences, in that no subject was classified into the A group by the AAFA model and the D group by the AFA model (Figure 2 and Multimedia Appendix 3). There were at most 2 levels of difference, as shown in Figure 2, indicated in red, green, and purple. After referring to the actual rate of poor ovarian response in these groups, we came to the conclusion that the 2 models have their own benefits and can complement each other in assessing ovarian reserve. Integration of these 2 models might give infertility clinics more individualized recommendations before starting controlled ovarian stimulation.

The global infertility rate is increasing, affecting about 1 in 7 couples [17]. A large proportion of women worldwide choose to delay having their first child for pursuit of opportunities to improve their education and workforce participation. It has long been acknowledged that fertility (the ability to establish a clinical pregnancy) decreases with increasing female age. Thus, the prevalence of infertility is increasing worldwide due to the postponement of childbearing. However, many women of reproductive age are not aware of the existing large heterogeneity in ovarian reserve for the same age [18]. In response to the increasing of infertility rate, to achieve a successful pregnancy, an increasing number of couples seek for assisted reproductive treatment. However, not all couples will benefit from it, as the beneficial effect of assisted reproductive treatment is limited in women with diminished ovarian reserve or in women with premenopause [19,20]. If women with potential diminished ovarian reserve could evaluate their ovarian reserve status earlier, it might be possible to avoid the subsequent infertility problem. Our new AFA model provides better means for assessing ovarian reserve, so that women of childbearing age, especially those who hesitate to start a family, might be able to evaluate their ovarian reserve in time.

The circulating AMH concentration is well-correlated with the AFC, and it is considered to be the best predictor for an ovarian response [3,14,21,22]. However, it should be noted that AMH concentrations and AFC are not necessarily linked. The term “ovarian reserve” refers to the number of primordial follicles remaining in the ovarian cortex. AMH is secreted by immature granulosa cells in the gonadotropin-independent phase of follicular development, while the AFC reflects the later gonadotropin-dependent phase. For example, in patients with hypogonadotropic hypogonadism, AFC is undetectable because of the extremely low level of FSH, but such young patients can have a sufficient ovarian reserve, manifested by normal AMH levels and good pregnancy outcomes when undergoing assisted reproductive technology. In addition, some patients exhibit a diminished ovarian reserve and low AMH concentrations but have a satisfactory AFC. AMH gene knockout mice might help us to understand the underlying mechanisms in such patients. In these mice, diminished ovarian reserve induced by the absence of AMH leads to accelerated follicular activation and an increase in the AFC in 4-month-old AMH-null mice (young adult) [23]. Therefore, it is possible that the AMH concentration is a more accurate measure of the actual ovarian reserve than the AFC. Furthermore, the main effect of AMH level was 62.0% in our AAFA model, and 85.2% in the AFA model, meaning that this hormone is the best predictor of ovarian reserve among the existing indicators.

The relationship between AMH concentration and pregnancy outcomes has been investigated extensively [14,24-27]. Fertility is defined as the natural capability to establish a clinical pregnancy [28]. The most accepted predictor for fertility is the ovarian reserve. Within a certain range, the number of primordial follicles does not correlate well with fertility [6], but when the number falls below a certain threshold, as in the case of diminished ovarian reserve defined by our AAFA [6] or AFA models (Table 5), female fertility declines significantly. This might explain the relatively weak relationship between fertility and ovarian reserve. There is a large variation in the number of granulosa cells needed to maintain at least 1 healthy oocyte; however, if there are too few granulosa cells to support at least 1 healthy oocyte, pregnancy is not possible.

There were some limitations to our study. First, it had a retrospective and nonrandomized design. However, as one of the largest reproductive centers in China, there is no strict limit on our selection of patients, thus helping avoid selection bias among our study population. Therefore, our AFA model is relevant to daily clinical practice. Second, our AFA model divides the population into 60 subgroups (3×4×5) rather than the 16 subgroups in the AAFA model. Thus, the sample sizes in our groups were relatively small, such as the 20th group (Multimedia Appendix 2) with only 1 case. We aim to include more samples in the future to verify and improve our formula used in the AFA model. Our last concern is that the positive rate predicted by the validation set (2018 data) is lower than the training set (2017 data), which may be induced by the lower rate of actual poor responders in 2018 data (315/1523 in 2017 data vs 499/3273 in 2018 data). Although the predicted positive rate of the validation set is low, considering the similarity of the AUC of the training set and the validation set, and the main purpose of our research, which is to classify the whole population into more groups according to the predicted probability of poor ovarian response, we believe that the AFA model is satisfactory and comparable to AAFA model. For subsequent related software, we will also integrate the AFA model, the AAFA model, and the actual rate of poor ovarian response in each subgroup together to further optimize the algorithm of this ovarian reserve assessment–related software.

## Appendix

Multimedia Appendix 1 A calibration plot of the AFA model in the training set and validation set.

Multimedia Appendix 2 Ranking the ovarian reserve into 4 subgroups of A-D based on predicted probability of poor ovarian response in validation data.

Multimedia Appendix 3 Raw data and corresponding predicted groups and subgroups classified by the AFA or AAFA models.

## Abbreviations

AFA: Anti-Müllerian hormone level–Follicle-stimulating hormone level–Age
AFAA: Anti-Müllerian hormone level–Antral follicle count–Follicle-stimulating hormone level–Age
AFC: antral follicle count
AMH: anti-Müllerian hormone
AUC: area under the curve
FSH: follicle-stimulating hormone
GnRH: gonadotropin-releasing hormone
NPV: negative predicative value
PPV: positive predictive value
