# Supplementary figures and images for: An Ovarian Reserve Assessment Model Based on Anti-Müllerian Hormone Levels, Follicle-Stimulating Hormone Levels, and Age: Retrospective Cohort Study
Source: J Med Internet Res. 2020 Sep 21;22(9):e19096. doi: 10.2196/19096 (PMC7546624; doi:10.2196/19096)

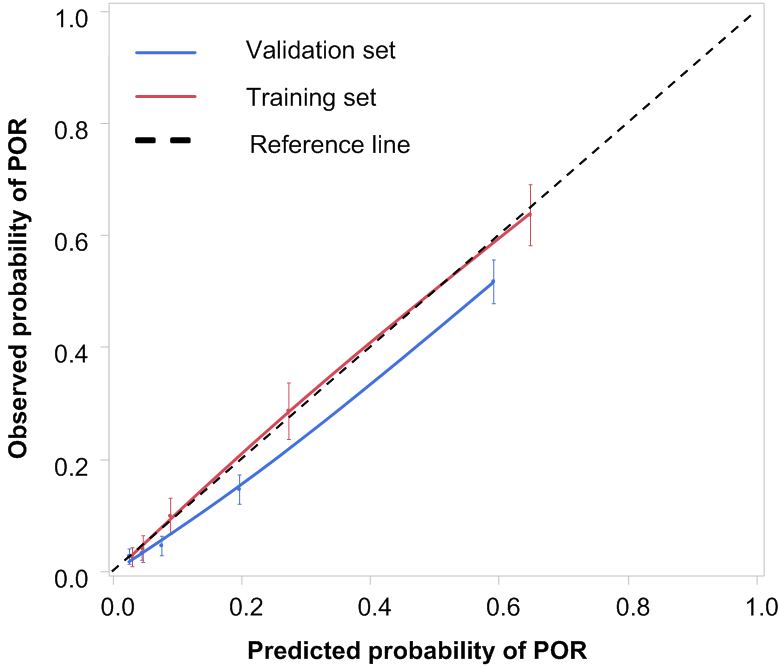

Supplement: Multimedia Appendix 1 [file jmir_v22i9e19096_app1.png]
