# Supplementary material for: An Ovarian Reserve Assessment Model Based on Anti-Müllerian Hormone Levels, Follicle-Stimulating Hormone Levels, and Age: Retrospective Cohort Study
Source: J Med Internet Res. 2020 Sep 21;22(9):e19096. doi: 10.2196/19096 (PMC7546624; doi:10.2196/19096)
Supplement: Multimedia Appendix 2 [file jmir_v22i9e19096_app2.docx]

**Multimedia Appendix 2.** Ranking the ovarian reserve into 4 subgroups of A-D based on predicted probability of poor ovarian response in validation data.

| **A** | | | | | | **B** | | | | | | **C** | | | | | | **D** | | | | | |
| --- | --- | --- | --- | --- | --- | --- | --- | --- | --- | --- | --- | --- | --- | --- | --- | --- | --- | --- | --- | --- | --- | --- | --- |
| **Ranked ovarian reserve** | **n** | **Predicted prob. of POR** | **Age group** | **FSH group** | **AMH group** | **Ranked ovarian reserve** | **n** | **Predicted prob. of POR** | **Age group** | **FSH group** | **AMH group** | **Ranked ovarian reserve** | **n** | **Predicted prob. of POR** | **Age group** | **FSH group** | **AMH group** | **Ranked ovarian reserve** | **n** | **Predicted prob. of POR** | **Age group** | **FSH group** | **AMH group** |
| 1 | 549 | 0.026 | 0 | 0 | 4 | 12 | 20 | 0.1 | 2 | 1 | 4 | 30 | 76 | 0.305 | 1 | 1 | 2 | 43 | 9 | 0.519 | 2 | 3 | 2 |
| 2 | 525 | 0.045 | 1 | 0 | 4 | 13 | 10 | 0.117 | 2 | 0 | 3 | 31 | 3 | 0.323 | 2 | 3 | 3 | 44 | 12 | 0.524 | 0 | 3 | 1 |
| 3 | 298 | 0.047 | 0 | 1 | 4 | 14 | 35 | 0.118 | 0 | 0 | 2 | 32 | 12 | 0.325 | 0 | 3 | 2 | 45 | 31 | 0.536 | 1 | 0 | 0 |
| 4 | 46 | 0.056 | 0 | 0 | 3 | 15 | 7 | 0.12 | 0 | 2 | 3 | 33 | 73 | 0.349 | 1 | 0 | 1 | 46 | 13 | 0.551 | 0 | 1 | 0 |
| 5 | 17 | 0.057 | 2 | 0 | 4 | 16 | 5 | 0.121 | 2 | 2 | 4 | 34 | 27 | 0.351 | 1 | 2 | 2 | 47 | 47 | 0.552 | 1 | 2 | 1 |
| 6 | 72 | 0.058 | 0 | 2 | 4 | 17 | 27 | 0.144 | 1 | 3 | 4 | 35 | 13 | 0.359 | 2 | 1 | 2 | 48 | 15 | 0.561 | 2 | 1 | 1 |
| 7 | 350 | 0.08 | 1 | 1 | 4 | 18 | 59 | 0.162 | 1 | 1 | 3 | 36 | 17 | 0.363 | 0 | 1 | 1 | 49 | 10 | 0.596 | 2 | 0 | 1 |
| 8 | 13 | 0.088 | 0 | 3 | 4 | 19 | 10 | 0.175 | 0 | 3 | 3 | 37 | 14 | 0.397 | 0 | 0 | 0 | 50 | 8 | 0.603 | 0 | 2 | 0 |
| 9 | 63 | 0.094 | 1 | 0 | 3 | 20 | 1 | 0.177 | 2 | 3 |  | 38 | 14 | 0.407 | 2 | 0 | 1 | 51 | 7 | 0.612 | 2 | 2 | 1 |
| 10 | 118 | 0.097 | 1 | 2 | 4 | 21 | 79 | 0.19 | 1 | 0 | 2 | 39 | 8 | 0.409 | 2 | 2 | 2 | 52 | 47 | 0.658 | 1 | 3 | 1 |
| 11 | 21 | 0.099 | 0 | 1 | 3 | 22 | 35 | 0.193 | 1 | 2 | 3 | 40 | 6 | 0.413 | 0 | 2 | 1 | 53 | 44 | 0.683 | 1 | 1 | 0 |
|  |  |  |  |  |  | 23 | 16 | 0.198 | 2 | 1 | 3 | 41 | 34 | 0.458 | 1 | 3 | 2 | 54 | 11 | 0.703 | 0 | 3 | 0 |
|  |  |  |  |  |  | 24 | 25 | 0.2 | 0 | 1 | 2 | 42 | 84 | 0.5 | 1 | 1 | 1 | 55 | 13 | 0.711 | 2 | 3 | 1 |
|  |  |  |  |  |  | 25 | 15 | 0.229 | 2 | 0 | 2 |  |  |  |  |  |  | 56 | 31 | 0.727 | 1 | 2 | 0 |
|  |  |  |  |  |  | 26 | 19 | 0.231 | 0 | 0 | 1 |  |  |  |  |  |  | 57 | 9 | 0.733 | 2 | 1 | 0 |
|  |  |  |  |  |  | 27 | 10 | 0.234 | 2 | 2 | 3 |  |  |  |  |  |  | 58 | 11 | 0.773 | 2 | 2 | 0 |
|  |  |  |  |  |  | 28 | 17 | 0.236 | 0 | 2 | 2 |  |  |  |  |  |  | 59 | 65 | 0.806 | 1 | 3 | 0 |
|  |  |  |  |  |  | 29 | 15 | 0.272 | 1 | 3 | 3 |  |  |  |  |  |  | 60 | 32 | 0.841 | 2 | 3 | 0 |
